# Flexible and Ecological Cotton-Based Dosimeter for 2D UV Surface Dose Distribution Measurements

**DOI:** 10.3390/ma17174339

**Published:** 2024-09-02

**Authors:** Elżbieta Sąsiadek-Andrzejczak, Piotr Maras, Marek Kozicki

**Affiliations:** 1 Department of Mechanical Engineering, Informatics and Chemistry of Polymer Materials, Faculty of Materials Technologies and Textile Design, Lodz University of Technology, Żeromskiego 116, 90-543 Lodz, Poland; 2 Department of Radiotherapy Planning, Copernicus Hospital, Pabianicka 62, 93-513 Lodz, Poland; piotr.maras@wp.pl

**Keywords:** flexible dosimeter, UV radiation, dose distribution, surface dose, technical textiles, cotton textiles

## Abstract

This work presents a 2D radiochromic dosimeter for ultraviolet (UV) radiation measurements, based on cotton fabric volume-modified with nitroblue tetrazolium chloride (NBT) as a radiation-sensitive compound. The developed dosimeter is flexible, which allows it to adapt to various shapes and show a color change from yellowish to purple-brown during irradiation. The intensity of the color change depends on the type of UV radiation and is the highest for UVC (253.7 nm). It has been shown that the developed dosimeters (i) can be used for UVC radiation dose measurements in the range of up to 10 J/cm^2^; (ii) can be measured in 2D using a flatbed scanner; and (iii) can have the obtained images after scanning be filtered with a medium filter to improve their quality by reducing noise from the fabric structure. The developed cotton–NBT dosimeters can measure UVC-absorbed radiation doses on objects of various shapes, and when combined with a dedicated computer software package and a data processing method, they form a comprehensive system for measuring dose distributions for objects with complex shapes. The developed system can also serve as a comprehensive method for assessing the quality and control of UV radiation sources used in various industrial processes.

## 1. Introduction

Ultraviolet (UV) artificial radiation is used in many industrial fields due to its usefulness in drying, sterilization, and detection applications. For this purpose, fluorescent lamps, tungsten, and quartz halogen filament bulbs are used. Various industries use a wide range of UV radiation, including UVA, UVB, and UVC ranges. In some cases, the intensity of UV radiation from some artificial sources may be similar to solar radiation. In printing applications, UV radiation is a technique used for rapid drying, heating, and curing inks, coatings, and layers, leading to improved productivity and print quality [1,2,3]. In the textile industry, UV radiation is used to dry dyed and refined products in order to improve the fabric’s colorfastness and durability [4,5]. In the cosmetics and healthcare industries, UV radiation is used for surface and tool sterilization, and purifying air and water [6,7,8]. Aesthetic cosmetics also use radiation during tanning, nail styling, dental treatments, and medical treatments like phototherapy for skin conditions such as psoriasis, vitiligo, and eczema [9,10,11,12,13,14]. Increasingly, radiation purification methods for water and air can be found in home solutions for people suffering from gastrointestinal diseases and allergies [15,16]. Irradiation with appropriate doses allows water and air to be purified from bacteria, viruses, allergens, and other pathogens, which is also useful in the food, beverage, and electronics industries [17,18,19,20]. Radiation processes also ensure the appropriate quality of products and packaging, which extends the shelf life of products and ensures the safety of their use. Moreover, in the case of packaging, it is possible to protect products against counterfeiting by using markers, e.g., in the form of paints that glow in a specific range of UV radiation [21,22]. In addition to recognizing the originality of products, UV radiation is also used in forensics to search for biological traces as evidence [23,24]. The examples of the technical use of UV radiation described above concern the distribution of radiation doses on flat, two-dimensional (2D) surfaces. However, 2D methods for verifying UV doses, e.g., using flat and stiff dosimetric films, do not provide objective results in the case of UV monitoring on objects with various, more complex structures and shapes.

In the case of radiation processes, the effectiveness of, e.g., phototherapy treatment is determined by the distribution of UV radiation, which ensures the patient’s safety and guarantees the expected effects [9,10,11,12,13,14]. This applies especially to processes in the fields of medicine and aesthetic treatments, where the precise setting of the radiation field has a direct impact on the patient’s safety. For example, in medical settings where UV radiation is used for phototherapy treatments, the distribution of UV radiation is monitored to ensure uniform exposure to the skin. Moreover, to maximize treatment effectiveness and reduce the risk of overexposure, UV dosimeters can measure the UV dosage that the patient receives during the treatment session. It is also worth mentioning that inappropriate UV irradiation of humans may lead to several complications, such as (i) skin aging and burns; (ii) eye damage; (iii) DNA damage; (iv) immune disorders; and (v) cancer and tumors [25,26,27,28]. In the case of printing techniques (traditional and 3D methods), controlling the distribution of UV radiation ensures proper curing of inks and coatings [29,30]. Similar to the electronics and chemical industries, uniform UV exposure across electronic components and substrates prevents materials from under- or over-curing [31]. Considering the above methods of using UV radiation, the dose distribution should be controlled using electronic meters and dosimeters, which can precisely determine the radiation dose distribution in two- and three-dimensional space (2D and 3D) on the surface of the object. Interestingly, there are no clearly defined measurement standards for monitoring the distribution of UV doses in the above-described applications. The International Commission on Non-Ionizing Radiation Protection (ICNIRP) and the World Health Organization (WHO) recommend measuring the Standard Erythema Dose (SED) to determine the radiation dose from artificial UV radiation sources. For example, the value of 5 SED corresponds to exposure to UV radiation at a dose of 50 mJ/cm^2^, and the maximum annual exposure should not exceed an erythemal-weighted dose of 150 SED (1500 mJ/cm^2^) for white-skinned people, according to Fitzpatrick’s scale [32,33]. Therefore, the most common methods for determining radiation doses are (i) electronic meters [34]; (ii) actinometers and photodiodes [35]; (iii) sensors based on inorganic materials [36]; (iv) water and alcohol solutions of photoluminescent dyes [37]; (v) liquid crystal mixtures [38]; or (vi) biological dosimeters [39]. However, the biggest disadvantage of these methods and devices is the inability to measure the distribution of UV radiation in 2D space. In medical applications, measurements of UV radiation are also limited to the use of 2D dosimeters, which can only determine the dose in the irradiation field and not its distribution on the 3D objects. A better solution is to use flat polymer films doped with UV-sensitive compounds, e.g., dyes, which change their color visible to the naked eye as a result of the absorbed dose of radiation. However, due to their stiffness and inability to bend or form on 3D objects, these films may lose information about the absorbed UV radiation dose. At this time, no literature reports have been found regarding the flexible, bendable dosimeters used in the above-discussed examples.

Previous works have shown that 2D polymer films doped with tetrazolium salts, 2D flexible flat textiles modified with gels on the surface [40,41], printed woven fabrics [42], and fibers doped with 10,12-pentacosadiynoic acid in polyacrylonitrile show potential for monitoring UVA, UVB, and UVC radiation doses. Textile dosimeters with nitroblue tetrazolium chloride (NBT) could also be suitable for 2D assessment of UV radiation [43] and ionizing radiation [44] distribution. Such dosimeters change color from a pale yellow to brownish-purple under UV or ionizing radiation exposure. The intensity of their color increases with the absorbed dose of radiation. For samples printed with NBT Pluronic F-127 water-based paste, it was shown that these samples have the greatest sensitivity to UVB radiation, and their dose sensitivity increases with an increase in NBT concentration. The characteristics of such a system printed on cotton fabric for a sample containing 2 g/dm^3^ of NBT include (i) a dynamic dose range equal to 0.01–3 J/cm^2^; (ii) a linear dose range equal to 0–3 J/cm^2^; (iii) a threshold dose of 0.01 J/cm^2^; and (iv) a dose sensitivity of 0.0719 ± 0.0016 cm^2^/J [43]. Such textile dosimeters are more flexible than commercially used dosimetric films for UV radiation measurements. Although they cannot be stretched, bending on the 3D surface does not result in loss of information about the absorbed dose. In addition, such a textile product may be subject to standard clothing and, e.g., be part of protective clothing for workers exposed to UV radiation. Additionally, the advantage of textile dosimeters is the ability to produce high volumes using commercially available machinery intended for the production of standard textile products. With appropriately established conditions for the manufacture, storage, and use of such dosimeters, this leads to better availability of such systems for UV radiation measurements and reduces manufacturing costs. However, samples made with the padding–squeezing–drying method can also be used for 2D dose distribution measurements of ionizing radiation due to the large dose range and high dose rates. It should be emphasized that in this method, samples are prepared from an aqueous NBT solution, and no other chemical modifiers are used. Therefore, using commonly used equipment for the chemical finishing of textiles, the production of such dosimeters is simple, quick, inexpensive, ecological, and adaptable to large production scales. 

The aim of this work was to develop a simple, chemically low-component, ecological 2D dosimeter based on a modified cotton woven fabric substrate and an aqueous solution of NBT as a radiation-sensitive color precursor for measuring the dose distribution of ultraviolet radiation. By using the padding–squeezing–drying method, it is possible to modify the volume of cotton fabric using a solution that only contains one radiation-sensitive compound, eliminating the need for additional surface modification techniques. Furthermore, this method will provide the dosimeter with appropriate elasticity and allow the production of larger amounts of material, even on an industrial scale. The dosimeter’s radiation reaction is based on the conversion of NBT into a colored formazan under ultraviolet radiation, which results in a color change from yellow to purple-brown. Based on previous research and experience with textile dosimeters, the following were investigated: (i) the dose–response of the dosimeter after irradiation with UVA, UVB, and UVC radiation; (ii) the assessment of color development in the CIELAB color system for calibration samples; (iii) scanning the textile dosimeter using a flatbed scanner and a dedicated computer software package as a dosimetry tool; (iv) the assessment of the possibility of 2D dose distribution measurements after inhomogeneous irradiation; and (v) the possibility of using the developed dosimeter for UV dose distribution measurements on non-flat surfaces. Based on the data obtained, the parameters characterizing the developed dosimeter were determined, and the strengths and weaknesses of the cotton–NBT system and the possibilities of its application were discussed. 

## 2. Materials and Methods

### 2.1. Preparation of Cotton Fabric Samples

In this study, cotton textile samples were used. The characteristics of the samples are as follows: 100% cotton, white not brightened woven fabric, twill weave, warp: 240/dm, weft: 220/dm, thickness of 0.68 mm, and surface weight: 250 g/m^2^ (Royal TenCate, Pendergrass, GA, USA). Before use, the cotton fabric was washed to remove impurities in an aqueous bath containing 0.1% of a non-ionic surfactant (Rokafenol N8-P7, Boruta Zgierz, Poland). The washing time and washing liquor temperature were 30 min and 40 °C, respectively. After washing, the fabric was rinsed with water and distilled water to remove the dirt and surfactant; it was dried (40 °C for 120 min) and ironed to flatten since otherwise creases on the samples would deteriorate further modification of the samples with radiation-sensitive compound. 

The periodic method of modification of cotton samples was chosen in this work, where samples were made in two steps: padding-squeezing (E. Benz, Stuttgart, Germany) and drying. They were soaked in a 10% nitroblue tetrazolium chloride solution (NBT, M = 817.64 g/mol, Roth, Karlsruhe, Germany) and squeezed (clamp 45 kG/cm, rotation speed 4 m/min). The drying step occurred at 30 °C for 120 min. The weight of the cotton sample (10.0 ± 0.1 cm^2^) before chemical modification, after soaking, squeezing, and drying was 2.325, 5.483, 3.442, and 2.483 g, respectively. After preparation, the samples were protected from daylight and stored at room temperature. 

### 2.2. UV Irradiation

The modified cotton samples were irradiated in the UV cabinets (Analytik Jena GmbH+Co. KG, Jena, Germany) at three wavelengths corresponding to UVA (8 W, type F8T5 Blacklight, range: 315–400 nm; a peak at 369 nm, Hitachi, Japan), UVB (8 W, type G8T5E, range: 280–360 nm; a peak at 306 nm, Sankyo Denki, Tokyo, Japan), and UVC (8 W, type G8T5, 253.7 nm, Sankyo Denki, Tokyo, Japan). The distance between the UV source and the lower part of the cabinet was unchanged and amounted to 18 cm. A given UV dose (mJ/cm^2^) was delivered automatically using a built-in detector and the control system of the device. To irradiate a sample with a dose of 100 mJ/cm^2^ of UVA, UVB, and UVC, the radiation exposure time equaled 1.0, 1.1, and 1.7 min. Four approaches to irradiation were realized. The first approach corresponded to calibration irradiation, where 36 samples were irradiated in the dose range of 0–5000 mJ/cm^2^. The second involved exposing cotton fabric to UV radiation through different patterns printed on polyethylene terephthalate (PET) foil. The third approach involved the irradiation of cotton fabric on the surface of triangular, rectangular, and cylindrical objects. For the triangular object, a glass prism was used with 3 cm sides and 15 cm lengths. For the rectangular object, a cuboidal box made of lustrous acrylonitrile-butadiene-styrene (ABS) of 4 cm^3^ × 15 cm^3^ × 15 cm^3^ dimensions was used. For the cylindrical object, a glass tube of diameter 2.5 cm and a length of 15 cm was used. Therefore, the samples were irradiated at distances of 15 cm, 14 cm, and 15.5 cm from the UV source for triangular, rectangular, and cylindrical objects, respectively.

### 2.3. 2D Scanning

The dosimeters (modified cotton fabric with NBT solution) were scanned using a flatbed scanner (HP Scanjet G3010, Hewlett-Packard Company, Palo Alto, CA, USA) with the following settings: 75 dpi, brighten/darken option: 11 (brighten), −69 (shadows), 0 (intermediate shades), image sharpening: none, color adjustment: none (color saturation 100%), automatic color correction: none. All samples were scanned approximately 2 h after exposure. To avoid accidental exposure to sunlight or artificial light, they were covered with aluminum foil.

### 2.4. Data Processing

The bitmaps obtained after scanning the dosimeters with the HP Scanjet G3010 scanner were processed using the polyGeVero^®^-CT software package (v.1.2, GeVero Co., Lodz, Poland) [45]. The images were transformed into 2D dose distribution images after applying a calibration equation. These dose maps were also exported to the polyGeVero^®^ software package (v.2.0, GeVero Co., Lodz, Poland) for further analysis. From the images obtained after scanning the cotton–NBT samples, the following was done using the software package: (i) filtering the images after scanning the dosimeter; (ii) calibrating the dosimeter; (iii) converting the obtained results into a UV radiation dose distribution; (iv) creating 2D/3D maps with UV radiation dose distribution.

### 2.5. Color Coordinate Measurements

The color coordinates of the cotton–NBT dosimeter samples were measured using a light reflectance instrument (Spectraflash 300, DataColor, Rotkreuz, Switzerland). All measurements were performed with the same settings in the microMATCH (v.3.6 software, DataColor, Rotkreuz, Switzerland) under a standard illuminant D65 at an angle of 10°, with a resolution of 10 nm, and a measurement error of 0.1% according to a calibration procedure described elsewhere [46]. The CIE L*, a*, b* color coordinates were determined using the CIE Lab 1976 evaluation system in accordance with the ISO/CIE 11664–4 standard [47], where “L*” determines the lightness of color from black to white on a scale from 0 to 100, the “a*” value represents the color components on the green–red axis and the “b*” value represents the color components on the blue-yellow axis.

Selected cotton–NBT samples were also photographed using a Canon 50D (Canon Inc., Tokyo, Japan) digital camera. All photographs were taken under standard illuminant D65 (color temperature 6504K, Veri Vide F18T8/D65, Enderby, Leicester, UK) with the following settings: resolution 15.1 MPix, pixel size 4.69 µm, without flash, the kit lens EF-S 18–200 mm f/3.5–5.6.

### 2.6. Preparation of Samples for Irradiation with Ionizing Radiation

Cotton–NBT samples were prepared in the same manner as described in Section 2.1. Then they were homogeneously and heterogeneously irradiated using a linear accelerator (ELU 6-E Elektronika, Moscow, USSR) in the dose range of up to 80 kGy. Before irradiating the calibration samples of the cotton–NBT dosimeter, three commercial dosimetric systems were used: (i) graphite calorimetric dosimeter (HRRL, Lyngby, Denmark, RISO calibrated); (ii) alanine pellet dosimeters (EPR e-scan Bruker, Billerica, MA, United States, according to ISO/ASTM 51607:2013); and (iii) film dosimeters (B3 WINdose Dosimeters, average thickness: 0.0190 ± 0.0003 mm, GEX Corporation) to confirm dose rate and sample orientation relative to the accelerator. A detailed description of the irradiation conditions and geometry has been described elsewhere [44]. After the sample irradiation, they were scanned using a flatbed scanner as described in Section 2.3. The obtained images were further processed using the polyGeVero^®^-CT (v.1.2, GeVero Co., Lodz, Poland) software package. To analyze the images after scanning, the color change assessment in the RGB color system and filtering using the mean filter at various settings were used. Based on the results, the optimal settings were determined as follows: kernel size: 3, kernel unit: mm, kernel mode: 2D, iterations: 2, which allowed the image to be smoothed without losing its shape. The images obtained in this manner were transformed into 2D dose distribution images after applying the calibration equation. These 2D dose maps were exported to the polyGeVero^®^ software package for further analysis. A detailed description of image processing in the polyGeVero^®^ and polyGeVero^®^-CT software packages has also been described elsewhere [44,45].

## 3. Results and Discussion

### 3.1. Calibration of Cotton–NBT Dosimeter

**Figure 1:**
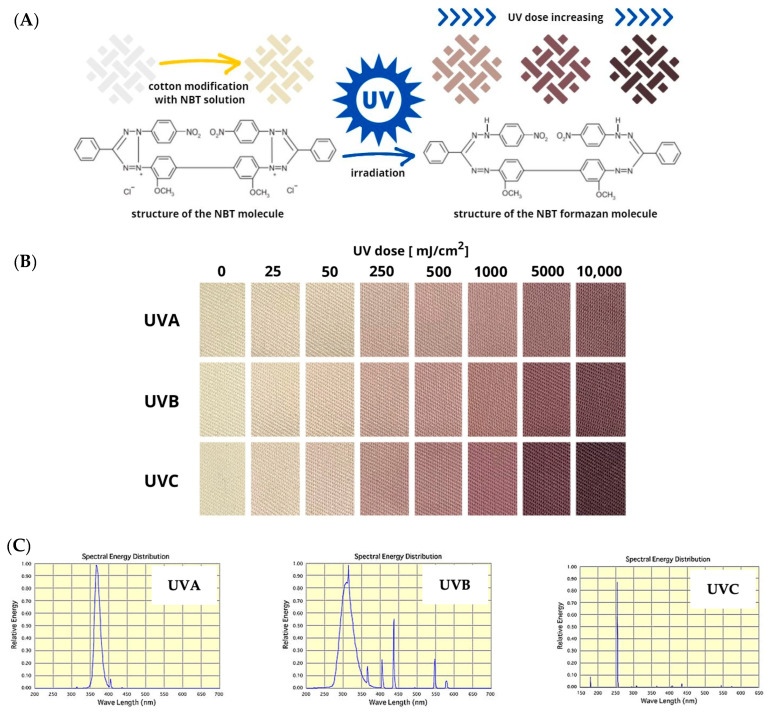
Color change of cotton–NBT dosimeters under the influence of UV radiation. A scheme of the transformation of the NBT molecule into colored formazan (**A**), the image of color changes for samples irradiated with UVA, UVB, and UVC radiation in the range of 0–10,000 mJ/cm^2^ scanned with a flatbed scanner (**B**) and spectral energy distribution graphs for UVA (368 nm), UVB (306 nm), and UVC (253.7 nm) lamps (**C**).

Cotton–NBT textile dosimeters change their color from light yellow to purple-brown when exposed to UV radiation. The intensity of the color change depends on the sub-range of UV radiation to which the sample is exposed and the absorbed dose of UV radiation. The higher the dose absorbed, the more intense the color of the sample (Figure 1A). Upon irradiation, the NBT molecule converts to formazan by dissociation of the N-N bond. The formation of formazan from various tetrazolium salts has been illustrated under different conditions elsewhere [48,49]. Bond dissociation energies for various compounds have been reported in several publications for simple inorganic and more complex organic molecules [50,51,52,53]. For instance, the dissociation energy of the N-N bond (N2 → 2N) is 941.69 kJ (0 K) and 945.42 kJ (298 K) in the gas state [51]. In turn, for C_6_H_5_NH-NH_2_, the bond dissociation energy is 167.36 kJ (298 K) [53] and for C_6_H_5_NH-NHC_6_H_5_, which is the closest to the chemical structure studied in this work, the bond dissociation energy is 92.05 kJ (298 K) [53]. It can also be observed that, depending on the type of UV radiation, for the same dose range, the character of color changes is similar, but in the case of UVC, a higher color intensity is visible than in the case of UVA and UVB (Figure 1B). The spectral energy distribution diagrams of the UVA, UVB, and UVC lamps used to irradiate the samples are presented in Figure 1C. Based on the above observations and the measurements of color coordinates in the CIE Lab color system described in Table 1, it was decided that only UVC radiation would be used in further experiments reported below.

**Figure 2:**
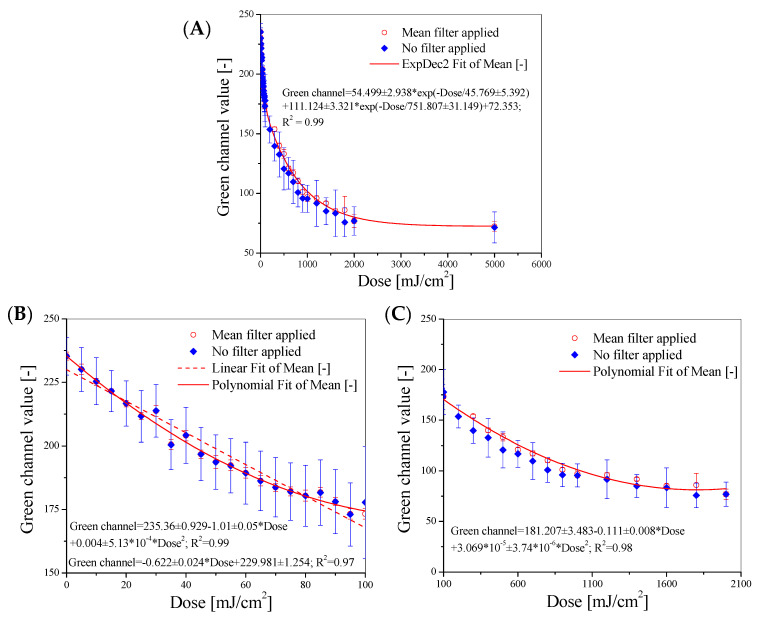
Calibration relations of the green channel values for RGB color model with respect to UVC dose absorbed by the samples, where (**A**) is for the full dose range, (**B**) is for the 0–100 mJ/cm^2^ dose range, and (**C**) is for 100–2000 mJ/cm^2^.

Comparing the lightness values of samples irradiated with a dose of 5000 mJ/cm^2^, differences of approximately 14%, 25%, and 36% can be seen for UVA, UVB, and UVC, respectively. First, calibration samples for UVC irradiation were prepared. A total of 36 samples were irradiated in the range of 0–5000 mJ/cm^2^ and scanned using a flatbed scanner. The cotton–NBT samples, as shown in the sample images after scanning in Figure 1B, have a structure that comes from the fabric’s weave. The surface texture is visible to the naked eye after scanning. Factors influencing the scanning of textile dosimeter samples using polyamide fabric and tetrazolium salts, including NBT, have already been analyzed in previous research [54]. It has been shown that features such as (i) type of raw material and weave of the fabric; (ii) scanning resolution in the range of 72–600 dpi; (iii) scanner control software settings, including the use of sharpening filters; and (iv) averaging of data after scanning have a large impact on the parameters characterizing the developed dosimeters for UV radiation measurements. Therefore, the samples were scanned with a resolution of 75 dpi, and no sharpening or color adjustment filters were used to improve the quality of the obtained image. The polyGeVero^®^-CT software package additionally processed the images of cotton–NBT samples after scanning to decompose them into color channels in the RGB color model. All channels significantly contribute to the color changes of irradiated samples, according to a comparison of the obtained images for the red, green, and blue channels. The color differences between non-irradiated and irradiated samples with a dose of 250 mJ/cm^2^ were 52.5, 59.1, and 40.3% for the red, green, and blue channels, respectively. On this basis, the green channel was selected as the one that most influenced the color change of the samples after irradiation. Then, using the tools of the polyGeVero^®^ software package, the images were filtered; the mean filter was applied to various settings. The following settings were used: kernel size: 3, kernel unit: mm, kernel mode: 2D, Iterations: 2. These settings allowed for smoothing the sample images without distortion in their dimensions and shapes. This procedure allowed the preparation of a calibration relation between the values of the green channel of the RGB color model and the absorbed dose, which is presented in Figure 2. Each measurement point in Figure 2A is the average value from the image of the entire sample, an area of 7000–10,000 points with standard deviation bars marked. Regarding the previously discussed issues with scanning textile dosimeters [54], the high standard deviation values are related to the noise generated by the cotton fabric’s weave structure. To reduce these values, the mean filter from the polyGeVero^®^ software package was used, and the obtained results are presented in Figure 2A.

The standard deviation values have been significantly reduced, and the points frequently cover the corresponding bars. Analyzing the response of the cotton–NBT 2D dosimeter, the following can be concluded: (i) the dynamic dose response is up to approximately 2000 mJ/cm^2^; (ii) the decrease in the value of the green channel as a function of the absorbed dose can be described by the function given in Figure 2A; however, the same relationship can be divided into two dose–response ranges: 5–95 mJ/cm^2^ and 100–2000 mJ/cm^2^, for which linear and polynomial calibration equations were obtained (Figure 2B,C); (iii) the dose sensitivity decreases as the purple-brown color conversion increases, becoming visible in the dose range above 100 mJ/cm^2^. Cotton–NBT samples, when stored away from sunlight or artificial light, are relatively stable. However, within 10 days, a change in the color of the samples by approximately 1.4% compared to the initial value was observed. Therefore, it is possible to use the developed dosimeters within several months of production, but this requires determining the exact operating conditions over a longer period of time, which this study did not assess.

### 3.2. Application—Rectangular, Cylindrical, and Triangular Objects

**Figure 3:**
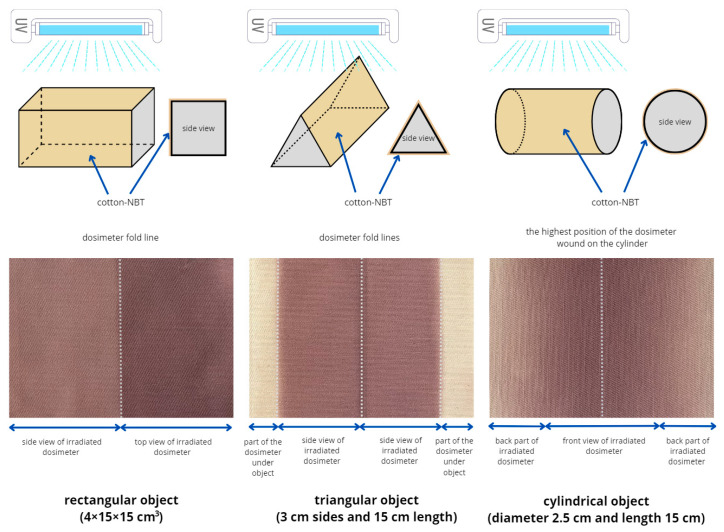
Scheme of UVC irradiation of cotton–NBT samples on rectangular, triangular, and cylindrical objects of various shapes (top line) and photographs of samples with visible effects of irradiation after removing them from these objects (bottom line).

In the next stage of the research, cotton–NBT samples were irradiated with UVC in a heterogeneous manner on three different surfaces of triangular, rectangular, and cylindrical objects, as shown in the scheme in Figure 3. In all three variants, the cotton–NBT sample was wound into an object and irradiated with a dose of 500 mJ/cm^2^. 

**Figure 4:**
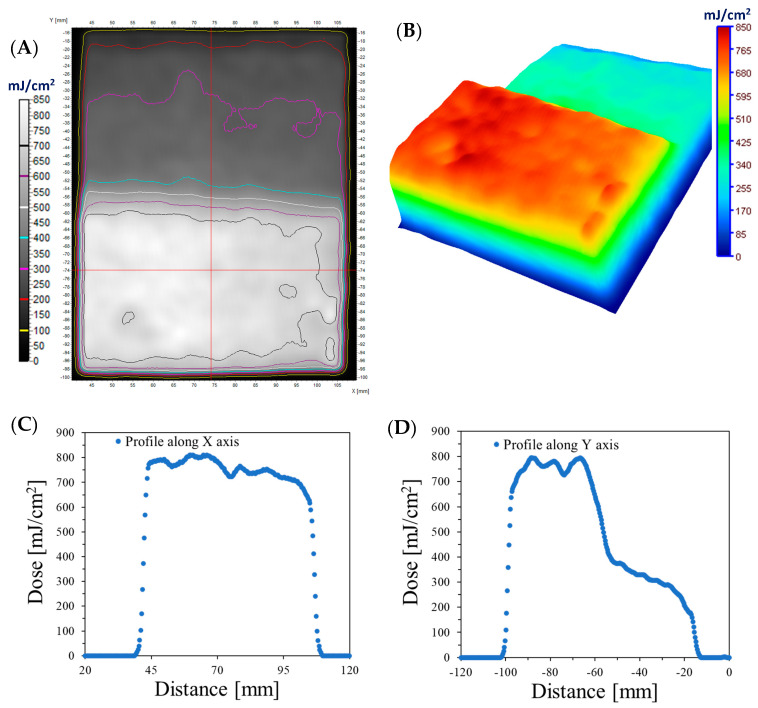
The results for the case where the cotton fabric dosimeter was placed on a box such that one part was on top of the box and the other part on a side of the box at a right angle and irradiated (dose emitted by the UVC radiator equaled 500 mJ/cm^2^); (**A**) is for the 2D absorbed dose map with isodoses superimposed; (**B**) is the same 2D dose map however illustrated in 3D using the Plane 3D option of the polyGeVero^®^ software package; (**C**,**D**) are profiles along the X and Y axes, respectively in the center of the dose distribution map in (**A**). The dose maps were calculated using the calibration relation given in Figure 2A.

The distribution of UVC radiation on the fabric surface affects the color of cotton–NBT samples removed after irradiation, depending on the shape of the used object. It should be added that the irradiated samples are relatively stable when stored at room temperature and covered with aluminum foil. It was calculated that after 30 days of storage of the samples, their color changed by approximately 7.2%. Thus, larger batches of dosimeters can be produced in one technological operation and stored in controlled conditions for a specified period of time, several days, weeks, or months without affecting the sample irradiation or reading after scanning. The topic of storage conditions for cotton–NBT dosimeters is not discussed in this work but is included in future work. For all three object shapes, the further processing results after irradiation were similar. The samples were removed from the object and scanned with a flatbed scanner, and the resulting images were processed as well as the calibration samples. All images were separated into RGB channels, and the green channel values were converted into dose values after applying the calibration relationship described in Section 3.1. In the case of a rectangular object, as a result of this operation, a two-dimensional map of the UVC radiation dose distribution was created, as shown in Figure 4. In addition, Figure 4A shows isodose lines superimposed on the dose distribution map. 

Figure 4B shows a dose distribution map using the dose distribution option in the 3D plane, additionally available in the polyGeVero^®^ software package. Figure 4C,D show two profiles along and across the irradiated areas, as shown in Figure 4A. The obtained results show that the maximum recorded radiation dose is approximately 750 mJ/cm^2^ in the part positioned perpendicular to the UVC radiation source. For a surface placed parallel to the UVC radiation beam, the maximum dose is approximately 800 mJ/cm^2^. The difference between the dose emitted by the device and the dose after processing in the software may be due to three reasons: (i) the use of a rectangular object with a lustrous, glossy surface (ABS) in the experiment, which can behave like a mirror and increase the absorption of UV radiation by the dosimeter; (ii) the sample’s distance from the UV source; and (iii) the unevenness of the cotton fabric results from the weave. Radiation reflected from the surface of lustrous, smooth ABS could have intensified the absorbed dose of UV radiation on the surface of the cotton–NBT dosimeter. The radiator’s built-in radiation detector sits approximately 1 cm above the irradiated surface. The use of a 4 cm high rectangular object reduced the distance of the sample from the light source, which increased the dose absorbed by the dosimeter by approximately 33%. The unevenness of the fabric weave, as described in Section 3.1, might be the cause of the difference between the emitted dose and the imaged dose. The use of the mean filter allows for improving the quality of the analyzed images, but at the same time averages the numerical values obtained from the irradiated field, taking into account the structure and non-uniformity of the textile substrate. The obtained results confirmed the possibility of using the modified cotton as a UVC radiation dosimeter for radiation measurements in various geometric planes. 

**Figure 5:**
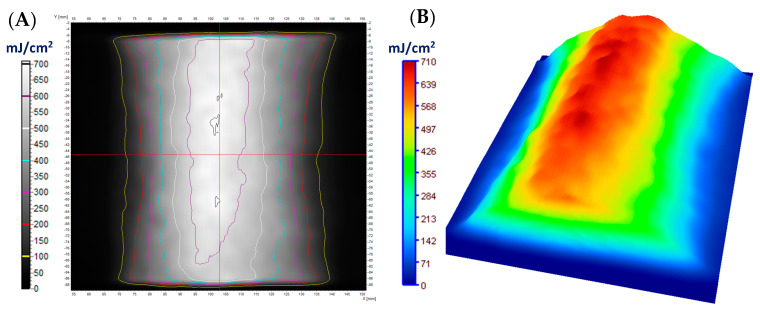

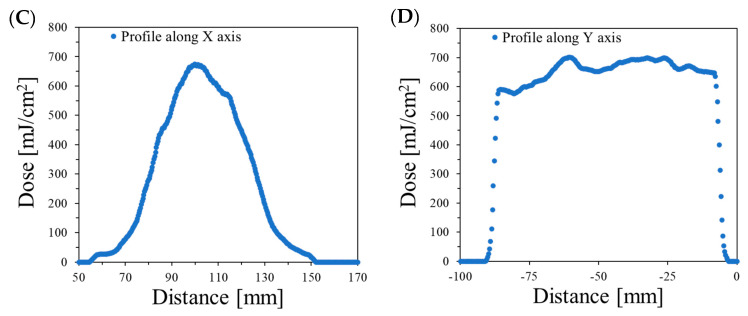
The results for the case where the cotton fabric dosimeter was wrapped on a cylindrical glass vial and irradiated (dose emitted by the UVC radiator equaled 500 mJ/cm^2^). (**A**) is for the 2D absorbed dose map with isodoses superimposed. (**B**) is the same 2D dose map, however, illustrated in 3D using the Plane 3D option of the polyGeVero^®^ software package; (**C**,**D**) are profiles along the X and Y axes, respectively in the center of the dose distribution map in (**A**). The dose maps were calculated using the calibration relation given in Figure 2A.

**Figure 6:**
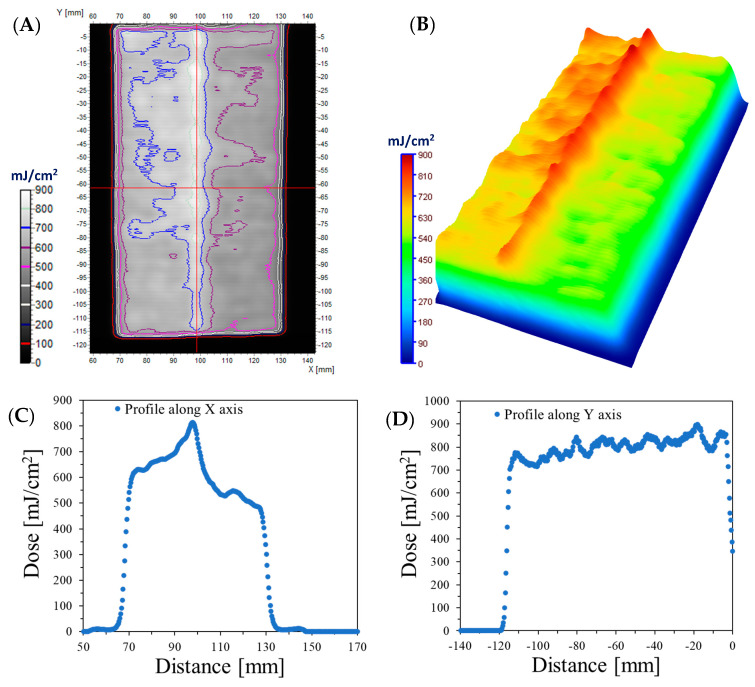
The results for the case where the cotton fabric dosimeter was wrapped on a triangular prism and irradiated (dose emitted by the UVC radiator equaled 500 mJ/cm^2^); (**A**) is for the 2D absorbed dose map with isodoses superimposed; (**B**) is the same 2D dose map, however, illustrated in 3D using the Plane 3D option of the polyGeVero^®^ software package; (**C**,**D**) are profiles along the X and Y axes, respectively in the center of the dose distribution map in (**A**). The dose maps were calculated using the calibration relation given in Figure 2A.

To demonstrate the possibility of using modified cotton as a flexible 2D dosimeter, a cotton–NBT sample was wound on a cylindrical object. After irradiation, the sample was removed from the cylinder and scanned, and the resulting images were processed as before. Figure shows that the sample’s color was the most intense in the central part, on an axis parallel to the UVC radiation source. From the front sides, it is visible that the color becomes less intense and almost disappears at the back, where the sample was attached to the cylindrical object. The visible gradient color fading on the sample may be caused by the reflection of UV radiation from the bottom of the device chamber. The chamber’s construction of brushed stainless steel shields it from dirt and simplifies cleaning, but it may also reflect a small amount of radiation. As in the case of rectangular objects, the distance between the cotton–NBT dosimeter and the radiation source is smaller because the cylinder used has a diameter of 2.5 cm. After irradiation, the sample was removed from the cylindrical object and scanned using a flatbed scanner. The resulting image was filtered using the mean filter and converted to a dose distribution as before, and the results of these operations are presented in Figure 5. Figure 5A shows a map of UVC radiation distribution with marked isodoses. Figure 5B shows a dose distribution map using the dose distribution option in the 3D plane, available in the polyGeVero^®^ software package. The obtained results show that the maximum recorded dose of UVC radiation is approximately 680 mJ/cm^2^. Figure 5C,D present exemplary profiles made along and across the cotton–NBT sample. The sample absorbed the highest dose in a line parallel to the radiation source. The color intensity of the sample decreases as the distance from this line increases sideways. A similar procedure was performed for the sample wrapped around a triangular object, and the results obtained are presented in Figure 6.

Figure 6A shows a map of UVC radiation distribution with marked isodoses for the cotton–NBT sample after irradiation, scanning, and image processing. Figure 6B shows a dose distribution map using the dose distribution option in the 3D plane, additionally available in the polyGeVero^®^ software package. The obtained results show that the maximum recorded dose of UVC radiation is approximately 800 mJ/cm^2^. Figure 6C,D, present exemplary profiles made along and across the cotton–NBT sample. As in the case of the cylindrical object, the cotton–NBT sample absorbed the highest dose in a line perpendicular to the radiation source. Additionally, the effect of increasing the radiation dose was also observed, as in the case of rectangular and cylindrical objects.

As a result of these experiments, the following conclusions can be drawn: (i) NBT-modified cotton fabric is suitable for measurements of 2D UVC dose distribution on the surfaces of various objects; (ii) the proposed image processing method, including image filtering, reduces image noise and allows to obtain high-resolution dose distribution maps; (iii) the differences between the emitted and absorbed radiation doses are related to the shape and geometry of the object’s surface; (iv) reducing the distance by 1 cm between the irradiated sample and the radiation source increases the absorbed dose by approximately 150 mJ/cm^2^; and (v) by combining the developed dosimeter with the scanning and data processing methods using polyGeVero^®^-CT polyGeVero^®^ software packages, it constitutes a comprehensive tool for 2D dosimetric analysis. Certainly, future research should also include: (i) measurements of the change in the dose absorbed by the dosimeter depending on the distance from the radiation source; (ii) the influence of the angle of incidence of UV light on the dosimeter surface; and (iii) UV transmittance through the modified textile substrate. Such a characterization was not within the research scope of the presented work, but it will certainly complement the previously obtained results regarding textile dosimeters.

### 3.3. Various Shapes of Irradiation

**Figure 7:**
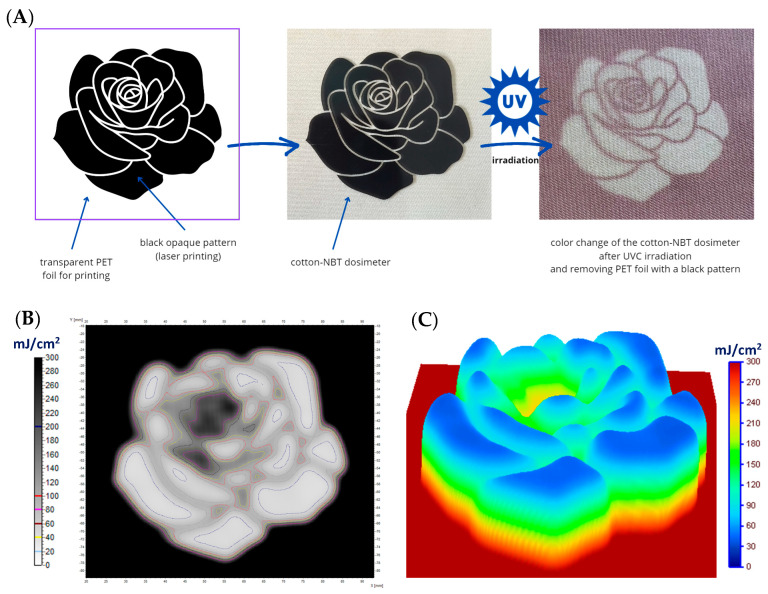
Scheme of preparing cotton–NBT for irradiation using an irregular pattern printed on polyester (PET) foil; (**A**) the results for the case where the cotton fabric dosimeter was covered with a pre-prepared pattern of a rose printed black on a transparent PET foil and irradiated (dose emitted by the UVC radiator equaled 5000 mJ/cm^2^); (**B**) is for the 2D absorbed dose maps with isodoses superimposed; (**C**) are the same 2D dose map, however, illustrated in 3D using the Plane 3D option of the polyGeVero^®^ software package. The dose maps were calculated using the calibration relation given in Figure 2B.

**Figure 8:**
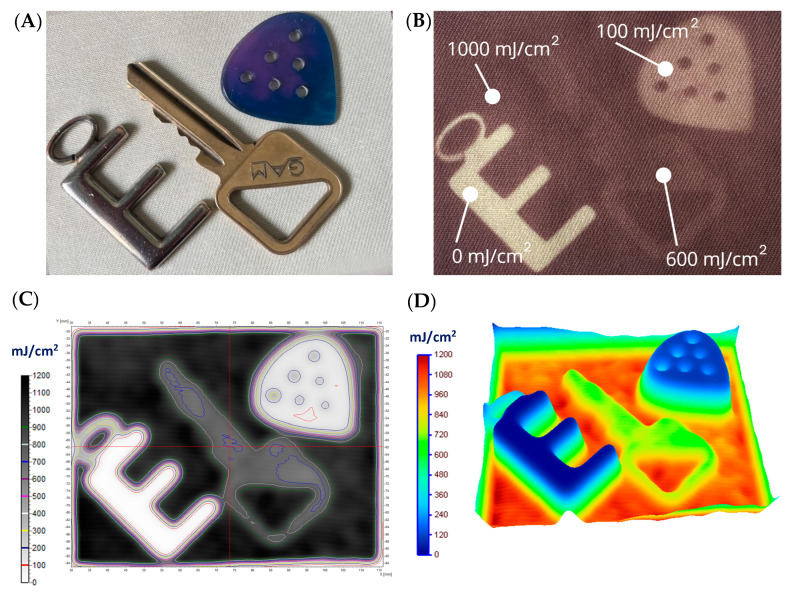
The results for the case where the cotton–NBT dosimeter was covered with three tools and UVC irradiated (**A**) (absorbed doses are marked in the sample photograph (**B**)); (**C**) is for the 2D absorbed dose map with isodoses superimposed; (**D**) is the same 2D dose map, however, illustrated in 3D using the Plane 3D option of the polyGeVero^®^ software package. The dose maps were calculated using the calibration relation given in Figure 2B.

In the next stage, cotton–NBT samples were prepared for non-homogeneous irradiation using irregular patterns. The first one was prepared in the form of a black pattern on polyethylene terephthalate (PET) foil, similar to the screen-printing technique. An opaque black pattern was printed on the foil using a laser printer, and the cotton–NBT sample was covered with it. The sample was then irradiated with UVC at a dose of 5000 mJ/cm^2^ (Figure 7A). After removing the foil, a white rose pattern was revealed on the surface of the modified fabric, which was covered with a black shape. It is worth noting that after irradiation, the color of the sample is much less intense than when a sample is not covered with PET foil. This effect occurs because the PET foil significantly reduces the UV radiation that is used to irradiate the cotton–NBT sample. Based on the calculations performed for the sample not covered and covered with PET foil, it follows that the difference in the absorbed dose by the sample is approximately 95%. These results were also confirmed after scanning the samples. Figure 7B shows a map of UVC radiation distribution with marked isodoses for the cotton–NBT sample after irradiation, scanning, and image processing. Figure 7C shows a dose distribution map using the dose distribution option in the 3D plane, available in the polyGeVero^®^ software package. The results show that the maximum recorded dose of UVC radiation is approximately 300 mJ/cm^2^. The use of a different foil substrate to create a black pattern was considered, but due to the laser printing technique, this is practically impossible. Commonly available printing materials in the form of transparent printing foils are usually PET. Their advantage is high uniformity and transparency, which do not cause unevenness during irradiation processes. Trials using other types of foil, such as polypropylene and polyethylene, made it impossible to print an irregular black pattern using the laser printer. Moreover, after irradiation, as in the case of PET foil, a reduction in the absorbed dose of UVC radiation was visible. Therefore, to show the possibility of recording different doses with the developed dosimeter, irregularly shaped objects were used instead of printed foil, as shown in Figure 8A. For this purpose, objects of various shapes (an e-shaped keyring made of 3 mm thick stainless steel; a metal key made of 1.5 mm thick brass, and a guitar pick made of 2 mm thick polylactide) were placed on the cotton–NBT surface and irradiated. To obtain the appropriate pattern, objects were removed from the sample surface after each dose. In this way, the areas were irradiated in the dose range of 0–1000 mJ/cm^2^, as shown in Figure 8B. Then, after UVC irradiation, the sample was scanned, and the resulting image was processed in the polyGeVero^®^-CT and polyGeVero^®^ software packages. Figure 8C shows a map of UVC radiation distribution with marked isodoses for the cotton–NBT sample after irradiation. Similarly to the previously described samples, a dose distribution map was also performed using the dose distribution option in the 3D plane (Figure 8D). The obtained data confirm that using the previously discussed procedure with the software packages, it is also possible to determine the non-homogeneous dose distribution using the developed cotton–NBT dosimeter.

Comparing the results obtained from the isodose map with the dose emitted by the UV source, a slight difference is visible in the case of the irregular shape of the metal key. On the sample surface and on the maps generated after scanning the sample, there are fewer details in the irradiated shape, which may be due to the raw material of the key and inaccurate pressing of it to the cotton–NBT surface. However, the conducted research concludes that the developed dosimeter can measure the dose and dose distribution of UVC radiation of a heterogeneous pattern.

### 3.4. Cotton–NBT Dosimeters for UVC vs. Ionizing Radiation—Discussion

**Figure 9:**
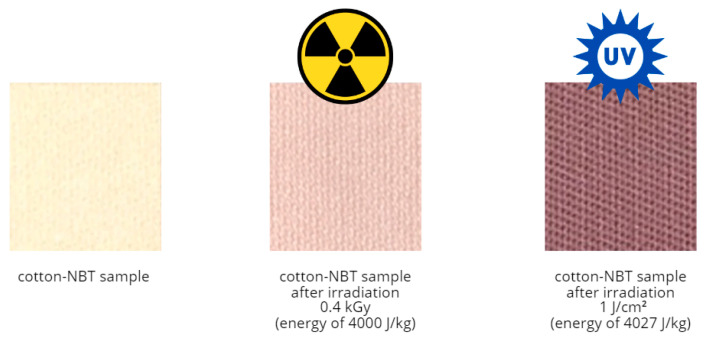
Comparison of the color difference of scanned cotton–NBT dosimeters after irradiation with the same energy value (4000 J/kg) of ionizing and UVC radiation.

The developed cotton–NBT dosimeters after irradiation with ionizing radiation showed the following characteristics: (i) the dose–response of the dosimeter is up to ~80 kGy; (ii) it is independent of the dose rate for 1.1–73.1 kGy/min; and (iii) two quasilinear dose subranges can be distinguished: ~0.6 to ~7.5 kGy and ~9.8 to ~61.9 kGy [44]. It should be noted that the research was carried out using only a technical electron accelerator and high doses of radiation, which are not applied in radiotherapy processes. The obtained research results encouraged further work on irradiating the developed system with other radiation sources, e.g., UV radiation. As a result of irradiation, the color changes are very similar for both types of radiation. The samples change from pale yellow to purple-brown as a result of the absorbed dose, and the color intensity increases as the radiation dose increases. However, in the case of UVC radiation, the color change is visible even at low doses. It is essential that an accelerator irradiates the sample in volume, whereas a UV source only irradiates the surface. As a result, it was important to determine the amount of energy that causes a color change in the cotton–NBT dosimeter after UVC irradiation and compare this value with the results obtained for ionizing radiation. Knowing that (i) the unit of measurement of the dose of ionizing radiation is gray (1 Gy = 1 J/kg); (ii) the unit of measurement of the UV radiation dose is 1 J/cm^2^; and knowing the mass of the irradiated cotton–NBT sample, it is possible to make a simple calculation and determine the amount of radiation energy that is delivered to the sample surface during UVC irradiation. The above relationship shows that the sample irradiated with 1 Gy was exposed to an energy of 1 J/kg, while the sample irradiated with UVC received an energy of 4027 J/kg (Figure 9). Thus, in the case of UVC radiation, the color change is visible to the naked eye even at a dose of 25 mJ/cm^2^, whereas in the case of ionizing radiation, it is slightly visible at a dose of 0.5 kGy. Undoubtedly, the presented research expands on the topic of cotton–NBT dosimeters for measuring doses, as well as the dose distribution of UVC and ionizing radiation.

### 3.5. Application of the Developed Dosimeters

**Figure 10:**
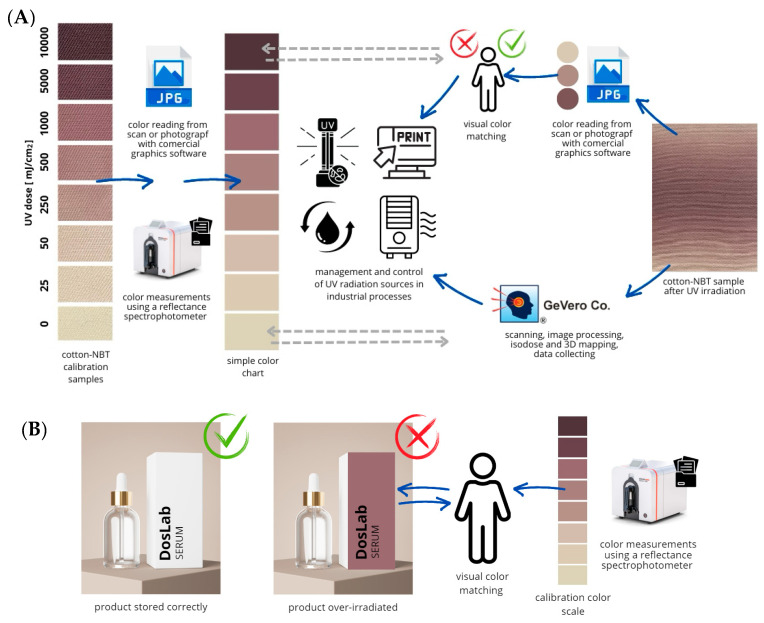
Connection diagram between visual assessment systems, color measurement, and 3D data processing in the management and control of industrial processes in which UV radiation is used (**A**) and an example method for assessing the correctness of storage of products in contact with UV radiation (**B**).

The proposed dosimeter, made of cotton fabric modified in volume with an aqueous NBT solution, is a quite simple and quick method of producing systems for measuring doses and dose distribution of UVC radiation. The proposed method of producing the dosimeter allows for the production of a large amount of such material without the need to use additional finishing agents. Therefore, it does not generate any environmentally harmful products that require proper storage or disposal. Consumer awareness is still growing in terms of environmental protection, and there may be objections to the use of cotton material to produce dosimeters. Controversies surrounding cotton include very high consumption of fresh water, use of large amounts of pesticides, depletion of land and insect populations, and unfair working conditions. Despite this, cotton is still at the top of the list of environmentally friendly fibers, among others, due to the possibility of reusing it after the recycling process. By using waste-water processes during cotton cultivation and in its processing, the production of dosimeters based on such fabrics should not result in additional production of waste that cannot be further processed. To reduce the impact of such a process on the environment, it is possible that similar dosimeters could be produced, e.g., in the form of paper modified with NBT or a different radio-sensitive ingredient. The proposed system for measuring dose and dose distribution on a 2D surface can constitute a comprehensive system for assessing the quality and control of UV radiation sources used in various industrial processes. Through comparative measurements and professional assessment in combination with dedicated software, one can control industrial processes in real time, making corrections on an ongoing basis and comparing them with the adopted, standardized calibration and control processes of such systems. Figure 10A presents an example of such management.

The proposed combination of a visual assessment system, color measurements, and 3D data processing is undoubtedly a comprehensive solution that does not fully address the issues related to the production of dosimeters based on textile materials. The developed dosimeters can also be successfully used as packaging elements for various products, e.g., cosmetics. Based on the color change, it could be determined whether a given product was exposed to excessive UV radiation during storage, reloading, or in-store conditions (Figure 10B). This research subject is not exhausted and can be continued in terms of the use of other UV-sensitive color precursors, various textile or paper substrates, as well as changes in the manufacturing and storage conditions of such dosimeters.

## 4. Conclusions

The work concerns the development of a 2D ecological, flexible dosimeter for measuring UVC radiation doses. The proposed dosimeter, based on a cotton fabric base modified in volume by the padding–squeezing–drying method, is simple to produce. The undoubted advantage of the developed dosimeter is the possibility of its production in large quantities using easily available, simple weaving machines, as well as standard wet processing and chemical finishing machines for processes used in the textile industry. The proposed method, with appropriately established conditions for the production, storage, and use of this type of dosimeter, may also lead to greater availability and lower costs of producing such systems for UV radiation measurements. The use of only an aqueous solution of nitroblue tetrazolium chloride as a radiation-sensitive compound limits the use of additional chemicals in the manufacturing process of these dosimeters. Deliberately limiting the necessary compounds in the developed chemical system is environmentally friendly and significantly reduces the financial outlays required to produce the dosimeter. It has been shown that the dosimeter responds to UV radiation in various subranges, and the color distribution is stable during storage. The proposed method of 2D scanning using a flatbed scanner combined with data processing using dedicated software packages allows for quick conversion of 2D images into 2D dose distribution after using the calibration function. The developed dosimeter can be used to measure 2D dose distribution in industrial processes using ultraviolet radiation and to monitor dose distribution for products of various shapes, e.g., packaging, and can be used as a complementary method in the control and monitoring of processes using UV radiation, e.g., printing processes or food packaging. The results discussed in the presented work do not exhaust the subject of textile dosimeters. Further research work may concern the following: (i) textile selection; (ii) changing the radiosensitive compound; and (iii) modifying non-textile substrates, e.g., paper. Based on the obtained results, future research should undoubtedly focus on the following: (i) measurements of the change in the dose absorbed by the dosimeter depending on the distance from the radiation source; (ii) the influence of the angle of incidence of UV light on the dosimeter surface; and iii) UV transmittance through the modified textile substrate. Such a characterization will certainly complement the previously obtained results regarding textile dosimeters.

## Figures and Tables

**Table 1:** Comparison of CIE L*, a*, b* color coordinates of cotton–NBT dosimeters irradiated with UVA, UVB, and UVC radiation in the dose range of 0–10,000 mJ/cm^2.^

UV Dose [mJ/cm^2^]	UVA			UVB			UVC		
	L*	a*	b*	L*	a*	b*	L*	a*	b*
0	83.45	−1.05	16.31	83.45	−1.05	16.31	83.45	−1.05	16.31
50	79.82	2.03	14.51	79.02	3.15	13.67	77.92	4.17	12.54
500	65.26	9.06	9.12	63.22	11.79	9.04	57.65	12.78	7.43
1000	59.27	11.77	8.02	55.70	13.87	8.83	48.10	16.63	4.23
5000	49.83	13.83	6.26	42.66	15.15	4.72	32.13	14.55	1.01
10,000	41.55	13.65	4.00	35.65	12.07	3.11	25.10	10.73	1.04

## Data Availability

The original contributions presented in the study are included in the article, further inquiries can be directed to the corresponding author/s.
